# Inhibition of CSF-1R Supports T-Cell Mediated Melanoma Therapy

**DOI:** 10.1371/journal.pone.0104230

**Published:** 2014-08-11

**Authors:** Marjolein Sluijter, Tetje C. van der Sluis, Pieter A. van der Velden, Mieke Versluis, Brian L. West, Sjoerd H. van der Burg, Thorbald van Hall

**Affiliations:** 1 Department of Clinical Oncology, Leiden University Medical Center, Leiden, the Netherlands; 2 Department of Immunohematology and Bloodtransfusion, Leiden University Medical Center, Leiden, the Netherlands; 3 Department of Ophthalmology, Leiden University Medical Center, Leiden, the Netherlands; 4 Plexxikon Inc., Berkeley, California, United States of America; Carl-Gustav Carus Technical University-Dresden, Germany

## Abstract

Tumor associated macrophages (TAM) can promote angiogenesis, invasiveness and immunosuppression. The cytokine CSF-1 (or M-CSF) is an important factor of TAM recruitment and differentiation and several pharmacological agents targeting the CSF-1 receptor (CSF-1R) have been developed to regulate TAM in solid cancers. We show that the kinase inhibitor PLX3397 strongly dampened the systemic and local accumulation of macrophages driven by B16F10 melanomas, without affecting Gr-1^+^ myeloid derived suppressor cells. Removal of intratumoral macrophages was remarkably efficient and a modest, but statistically significant, delay in melanoma outgrowth was observed. Importantly, CSF-1R inhibition strongly enhanced tumor control by immunotherapy using tumor-specific CD8 T cells. Elevated IFNγ production by T cells was observed in mice treated with the combination of PLX3397 and immunotherapy. These results support the combined use of CSF-1R inhibition with CD8 T cell immunotherapy, especially for macrophage-stimulating tumors.

## Introduction

Cells of the myeloid lineage are prominent components of the tumor microenvironment of solid malignancies and abundant infiltration of macrophages correlates with poor prognosis in most human cancers [1], [2], [3]. Activities of these tumor associated macrophages (TAM) that can explain a detrimental role include production of tumor growth factors, promotion of angiogenesis, creating invasive behavior through tissue remodeling and dampening cytotoxic immune responses. These characteristics of TAMs have been functionally linked to cancer progression and metastases by a series of mechanistic studies in mouse models [3]. The CSF-1 receptor (M-CSF receptor or CD115) is a key regulator for monocyte differentiation from progenitors of the bone marrow and also determines monocyte activation and migration [4]. Additionally, CSF-1R has been shown to polarize macrophages towards an immunosuppressive and tumor-promoting direction [5]. High levels of CSF-1 in blood of cancer patients is associated with poor prognosis and a common source of this cytokine is the tumor itself, thereby fostering a growth-supportive microenvironment. A second ligand for the CSF-1R, IL-34 could also conceivably regulate TAM. Considering this role of CSF-1R ligands and TAM, targeting agents for these cytokines and their receptor have been developed, including blocking antibodies and small molecule tyrosine kinase inhibitors. Currently, multiple clinical trials evaluate the safety and efficacy of these compounds in different types of cancer as single agents and as combination therapy [4].

We here studied the effects of CSF-1R inhibition in the context of CD8 T cell-mediated immunotherapy in the B16F10 mouse melanoma model. PLX3397 kinase inhibition was remarkably efficient in removing F4/80^+^ macrophages from the tumor site. Although single PLX3397 treatment only modestly delayed tumor growth, combination with tumor-specific CD8 T cells strongly promoted the control of tumor outgrowth, most likely through enhancement of T cell effector functions. Our data support further development of treatments combining immunotherapy with TAM targeting agents.

## Materials and Methods

### Mice, cell lines and reagents

C57BL/6jico mice were purchased from Charles River (Lille, France) and used at 8 to 10 weeks of age. Pmel-1 TCR transgenic mice (Thy1.1 background) harbor the gp100_25-33_/D^b^-specific receptor were bred and housed in the animal facility of the Leiden University Medical Center under specific pathogen-free conditions. Mice were kept in closed-controlled cage systems with food and water at libitum. Tumor grow experiments in mice were done with randomized female mice with five mice per cage. Tumor sizes were measure twice a week until tumors reached maximum 1000 mm^3^. Mice were sacrificed by cervical dislocation when tumors reached maximum size or lost more than 10% body weight or with unusual behavior as the result of suffering. Mice were monitored three times a week for welfare condition and potential discomfort. Maximum degree of discomfort was classified as low to moderate, therefore, no analgesics or anesthetics were applied. Experiments were approved by the local university committee for the care of laboratory animals (Dier Experimenten Commissie), in accordance with guidelines of the National Institutes of Health. B16F10 melanoma cell line was originally obtained from the American Type Culture Collection (CRL-6475) and were maintained in tissue culture for six months as described [6]. The CSF-1R kinase inhibitor PLX3397 incorporated into rodent chow at 290 mg/kg chow (delivering daily doses of approximately 45 mg/kg), was provided along with control chow by Plexxikon Inc [7], [8]. PLX3397 is a dual inhibitor of the CSF-1R and KIT kinases.

### Immunotherapy of B16F10 melanoma

A lethal dose of 3×10^4^ B16F10 melanoma cells was injected s.c. in syngeneic C57BL/6 mice. On day 3, PLX3397 or control chows were started and continued for the remainder of the experiment. Previously established protocol for transfer of pmel-1 T cells and vaccination with 20-mer long gp100 peptide was applied [6]. Tumor growth was monitored twice a week by measurement with a caliper in three dimensions.

### Flow cytometry

Blood samples and intratumoral immune cells were phenotyped by flow cytometry using panels of fluorescently-labeled antibodies on tumors that were resected and dissected using collagenase and DNAse [9]. Intracellular IFNγ measurements on CD8 T cells were performed after o/n stimulation with the mouse gp100 9-mer peptide [9]. Samples were acquired with Fortessa or Calibur flow cytometers (BD Biosciences) and analyzed with FlowJo software (TreeStar).

### Immunohistochemistry

Immunohistochemistry for the F4/80 macrophage marker was performed as previously described [10].

### Statistical analysis

Statistical analyses (Graph Pad Prism software) are indicated in the figure legends.

## Results and Discussion

### B16F10 melanomas promote the accumulation of macrophages in the circulation

**Figure 1:**
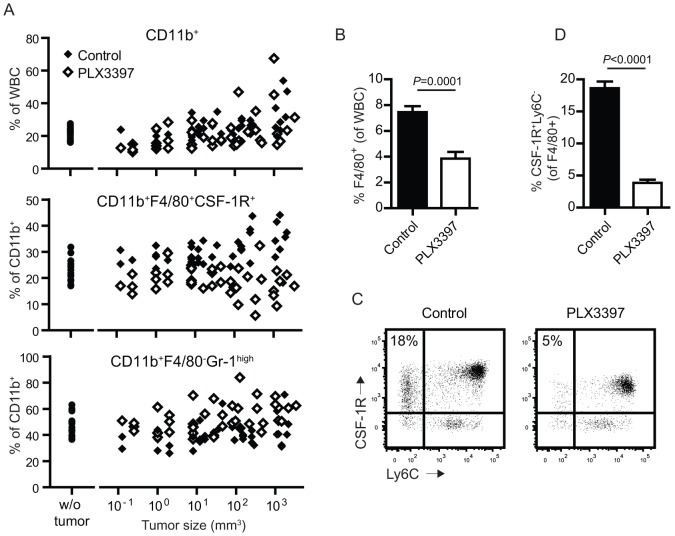
Systemic inhibition of CSF-1R decreases B16F10-induced accumulation of blood monocyte subsets. (A) Myeloid populations in the blood were monitored by flow cytometry following B16F10 tumor growth. Percentages of CD11b^+^ myeloid cells out of total white blood cells (WBC) and frequencies of CD11b^+^F4/80^+^CSF-1R^+^ monocytes or CD11b^+^Gr-1^high^ granulocytic cells within the CD11b^+^ population are depicted of control- and PLX3397-treated mice. Data are compiled from two different experiments with ten mice per group. Linear regression analysis indicated a significant difference in frequencies between control- and PLX3397-treatment measurements for all three graphs and a significant slope difference of CD11b^+^F4/80^+^CSF-1R^+^ cells (*p* = 0.04). (B) Frequencies of CD11b^+^F4/80^+^ monocytes in the blood as a proportion of total white blood cells at day 13 of B16F10 tumor growth. (C) Flow cytometry plots of blood CD11b^+^F4/80^+^ cells at day 13 of B16F10 tumor growth. (D) Frequencies of Ly6C^-^ cells within this gate are plotted for eight mice per group. Means and standard error of the mean of five mice per group are depicted from one out of two similar experiments.

We evaluated the composition of myeloid cells in blood and in B16F10 melanomas during the course of tumor growth. The overall proportion of myeloid cells in the blood modestly increased during tumor growth, indicating that the presence of the melanomas slightly enlarged the size of the circulating CD11b^+^ myeloid cell pool (Fig 1A, black symbols). Dissection of the myeloid cell subset by staining CSF-1R, F4/80, and Gr-1 molecules revealed that the relative proportion of blood monocytes (CD11b^+^F4/80^+^CSF-1R^+^Gr1^-^) doubled in the blood of mice bearing melanomas reaching 1 cm^3^ in size, whereas granulocytic myeloid cells (CD11b^+^F4/80^-^Gr-1^high^) increased much less. CSF-1R signaling is known to promote the differentiation of myeloid progenitors to macrophages and to regulate the migration and function of macrophages. We therefore treated tumor-bearing mice with the oral kinase inhibitor PLX3397 and observed a strong decrease of the tumor-induced F4/80^+^ monocytes in blood (Fig 1A and B) with concomitant increased frequencies of circulating granulocytic CD11b^+^Gr-1^high^ myeloid cells. This was likely a direct effect on hematopoietic cells, because B16F10 melanomas do not express CSF-1R (not shown). Detailed analysis on monocyte subsets in PLX3397-treated animals revealed two interesting findings. First, the inhibitor appeared to predominantly affect F4/80^+^Ly6C^-^ blood macrophages (Fig 1C and D), also described as ‘resident’ monocytes with functions for the vasculature [11],[12]. Second, the CSF-1R expression levels on F4/80^+^Ly6C^+^ ‘inflammatory’ monocytes was strongly decreased in PLX3397-treated mice (MFI 1772±479 versus 5813±551 in controls). The lower levels of CSF-1R might be the result of the small drug or, alternatively, these ‘inflammatory’ monocytes might represent more immature immigrants from the bone marrow. We concluded that the kinase inhibitor was capable to reduce frequencies of monocytes in the circulation in tumor-bearing mice.

### Inhibition of CSF-1R reduces intratumoral F4/80^+^ macrophages

**Figure 2:**
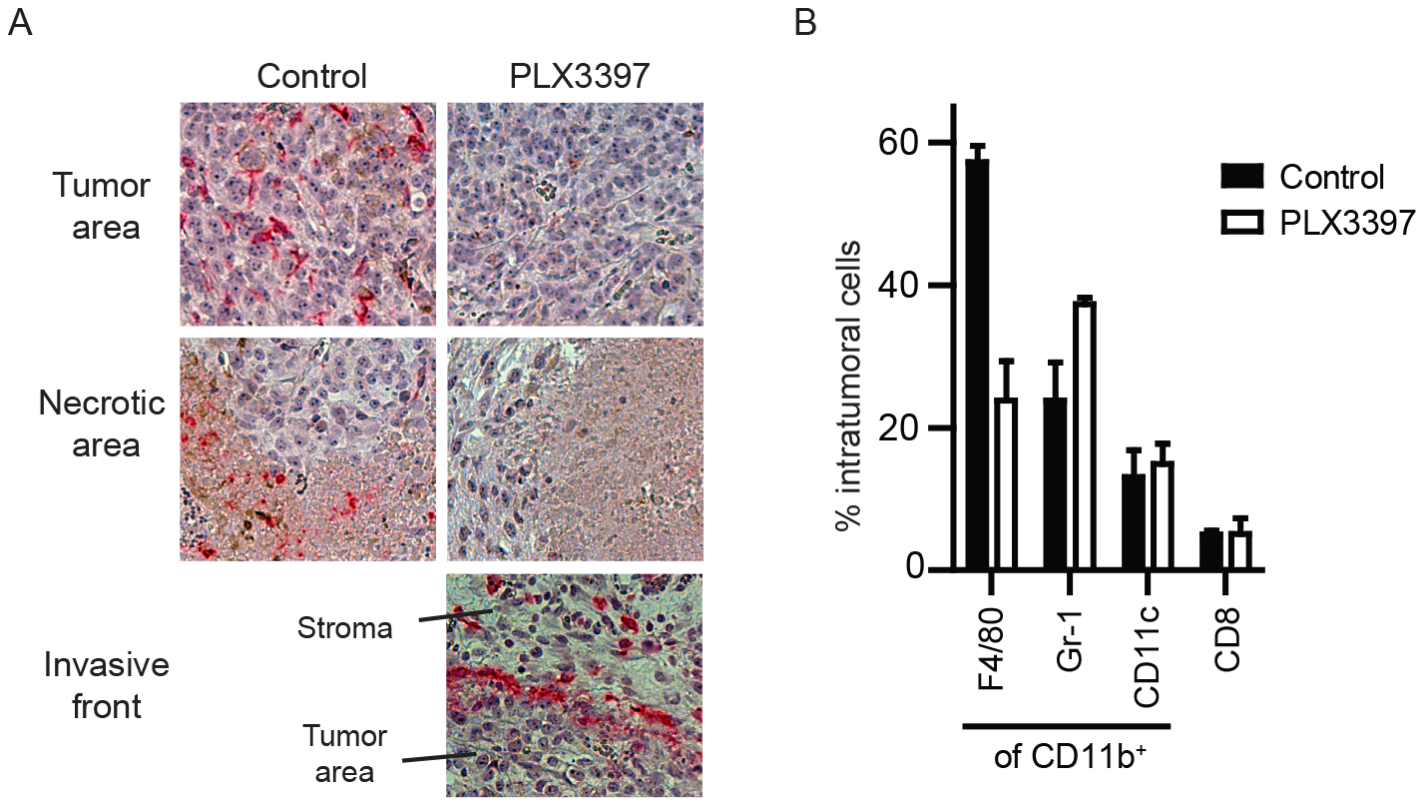
Decreased frequencies of tumor infiltrating macrophages. (A) B16F10 tumors were resected from mice at a size of 200 mm^3^, embedded in paraffin and stained for F4/80, as a marker for macrophages. Photos are representative for the distinct tumor areas and for more than ten analysed tumors. (B) Three tumors with comparable sizes were resected, dissociated to single cells and analysed by flow cytometry. Frequencies of myeloid subsets (from all CD11b^+^) are depicted. Frequencies of CD45^+^ infiltrating cells are depicted for CD8 T cells. Means and standard error of the mean are shown.

We then evaluated the presence of intratumoral macrophages and their sensitivity for the kinase inhibitor. Tissue sections of B16F10 tumors demonstrated a dense infiltration of F4/80^+^ macrophages (Fig 2A). Strikingly, tumors from mice systemically treated with PLX3397 lacked these macrophages, even in necrotic areas, which are typically endowed with F4/80^+^ cells. Flow cytometry data of dispersed B16F10 tumors confirmed the strong decrease of macrophages at the cost of increased proportions of Gr-1^+^ cells (Fig 2B). The residual macrophages that were detected in flow cytometry were located at the invasive front, as these were less efficiently removed by PLX3397 (Fig 2A). CD11b^+^CD11c^+^ myeloid cells in the tumor were not altered. Thus, inhibition of the CSF-1R removes tumor-infiltrating F4/80^+^ macrophages, in addition to their precursors in the circulation.

### Removal of intratumoral F4/80 macrophages delays tumor outgrowth

**Figure 3:**
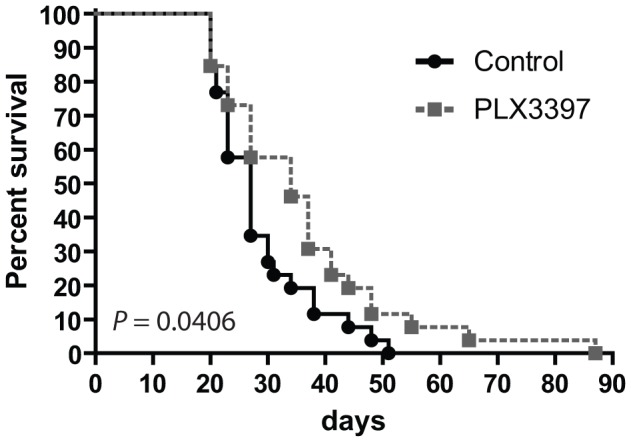
Macrophage reduction slows down melanoma outgrowth. Kaplan-Meier survival curves from control and PLX3397-treated mice are shown of 26 mice per group, compiled from three independent experiments. Mice were sacrificed when tumors reached 1000 mm^3^. Statistical analyses was performed with log-rank test.

We then examined B16F10 outgrowth during PLX3397 treatment. Kaplan-Meier analysis revealed a small but statistically significant survival benefit (*p* = 0.04) for mice treated with the CSF-1R inhibitor (Fig 3). These data suggested that macrophages harbored a tumor-promoting role in the B16F10 melanoma model. The rather small survival benefit of macrophage depletion surprised us, but might be explained by the relative increase of granulocytic myeloid cells, among which CD11b^+^Gr-1^+^ myeloid derived suppressor cells (MDSC) as found in many mouse tumor models [1], [13], [14]. Each tumor type sculpts their microenvironment in different directions and it remains to be evaluated which exact cues of the tumor drive myeloid differentiation pathways. We previously reported that tumor-secreted IL-6 and prostaglandin E2 are key in the differentiation to M2-type macrophages [15]. Nonetheless, a multitude of other soluble factors has been demonstrated to impose altered myeloid cell differentiation in the presence of solid tumors [1].

### PLX3397 enhances CD8-mediated immunotherapy of melanoma

**Figure 4:**
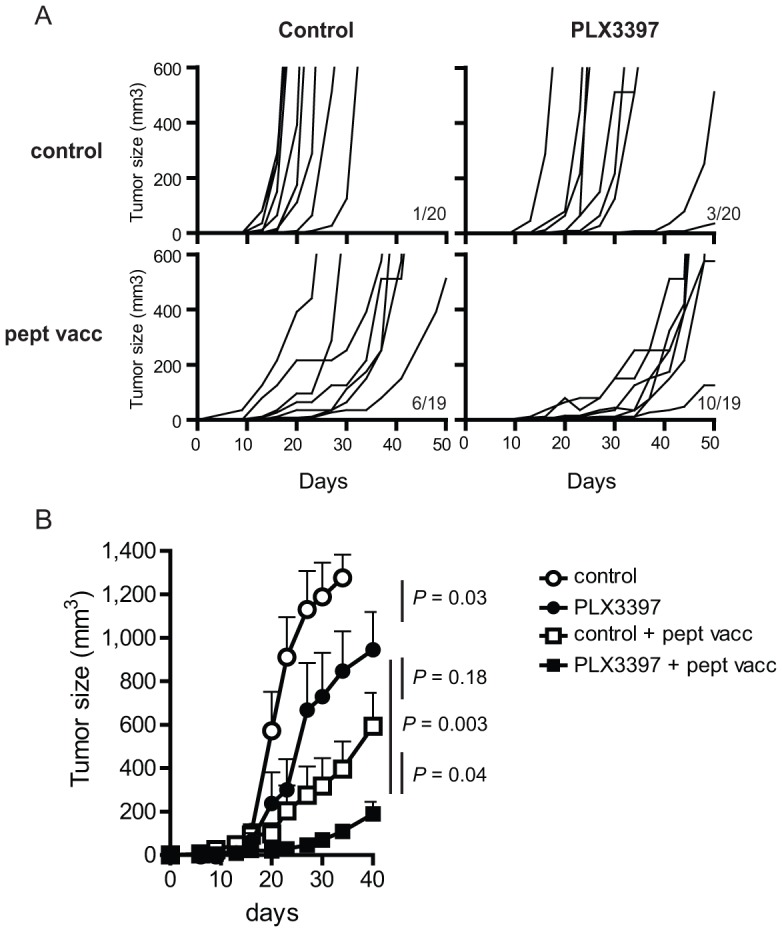
CSF-1R inhibition enhances CD8-mediated immunotherapy of melanoma. (A) Tumor growth curves of individual mice are shown for the four different treatment groups. Immunotherapy is designated as ‘pept vacc’ and contains transfer of pmel-1 CD8 T cells and peptide vaccination. Numbers in the panels indicate the group size and the number of mice that are still alive at day 50. This experiment was repeated once with comparable outcome and indicated survival rates are compiled from both. (B) Average tumor sizes and standard error of the mean per group from experiment shown in panel (A). Statistical analysis was performed with two-way ANOVA after Bonferroni correction.

These results prompted the application of CSF-1R inhibitors in combination therapy with immune-intervention. Previously, we demonstrated partial control of B16F10 tumor outgrowth by vaccination with long synthetic peptides [6], [9]. The transfer of naïve melanoma-directed CD8 T cells and subsequent *in vivo* activation by a long synthetic peptide vaccine resulted in massive expansion of the anti-tumor T cells and partial control of B16F10 melanomas [6]. Importantly, myeloablative host conditioning regimens were avoidable in this treatment scheme. Although we found that PLX3397 strongly diminished tumor macrophages, the natural influx of CD8 T cells to the tumor was not impaired (Fig 2B). This encouraged us to test treatments combining adoptive T cell transfer, peptide vaccination and PLX3397. Interestingly, CSF-1R blockade caused a small delay in the onset of tumor outgrowth, but without altering the doubling time of the tumors (Fig 4A). In contrast, immunotherapy resulted in a slower growth rate of the tumors, without affecting the time of their appearance. The combined treatment resulted in significantly delayed tumor growth that was superior to either of the single approaches (Fig 4B). Importantly, fifty percent of the mice treated with the combined therapy was still alive at day 50 (10 out of 19), whereas survival rates were much lower for the single approaches (Fig 4A). This slowdown of outgrowth was accompanied by a strong influx of tumor-specific CD8 T cells [6], [9].

**Figure 5:**
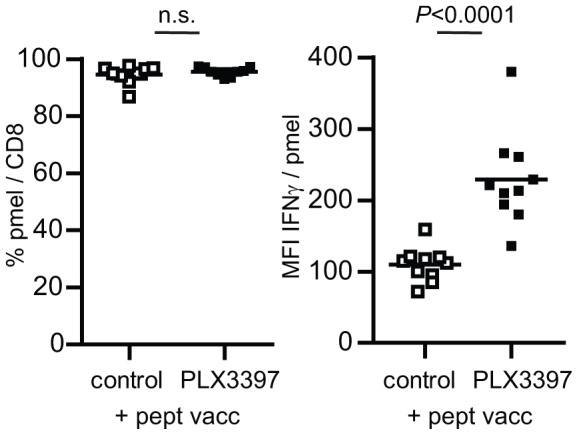
IFNγ production of tumor-specific CD8 T cells. Frequencies and IFNγ levels of transferred pmel-1 T cells were analysed in the blood at the peak of the response on day 21. MFI indicates intensity of IFNγ staining per pmel-1 population (geo-means). Two-way student t-test was used to calculate statistical significance.

In an attempt to delineate the critical factors responsible for the success of the combination therapy, we tested the frequency and activity of the anti-tumor pmel T cells. The frequency of pmel T cells in the blood was already high in the immunotherapy alone group and not altered (Fig 5). However, the level of INFγ-production per cell was significantly higher in mice that were treated by a combination of CSF-1R inhibition and CD8 T-cell based immunotherapy (Fig 5). The importance of INFγ in the immune control of tumors is widely described [16] and reduced presence of macrophages in the local tumor environment is likely to empower effector functions of intratumoral T cells. Immune suppressive activity of M2-type macrophages have been thoroughly investigated in mouse and human tumors and mechanisms of dampening T cell effector functions include nitrosylation of TCR, production of immunosuppressive cytokines IL-10 and TGFβ and amino acid deprivation [3], [14], [17].

## Conclusion

Pharmacological targeting of the CSF-1R is a subject of major interest in the oncology field. Several publications reported efficient depletion of M2-type tumor macrophages by blocking antibodies or specific tyrosine kinase inhibitors. Most of these compounds are capable of modest tumor growth delay in transplantable and spontaneous tumor models [8], [18], [19], [20], [21], [22]. Interestingly, CSF-1R inhibition in mouse glioblastoma was shown to result in macrophage polarization as witnessed by decreased M2-type effector molecules and strong survival benefit of treated animals [20]. In this latter case, the glioblastoma tumor cells secreted strong macrophage survival factors, preventing macrophage depletion and promoting their polarization. Currently, a dozen clinical trials are evaluating CSF-1R targeting compounds as therapeutic agents for malignancies. Our data show that these agents are suitable to be combined with immunotherapy and suggest that such combinatorial approaches might be particularly interesting for tumors that drive hematopoietic differentiation towards macrophages.
